# EMILIN2 is associated with prognosis and immunotherapy in clear cell renal cell carcinoma

**DOI:** 10.3389/fgene.2022.1058207

**Published:** 2022-12-05

**Authors:** Guangjian Zhao, Jianpei Zheng, Kai Tang, Qi Chen

**Affiliations:** Fujian Key Laboratory of Innate Immune Biology, Biomedical Research Center of South China, Fujian Normal University Qishan Campus, Fuzhou, China

**Keywords:** EMILIN2, clear cell renal cell carcinoma (ccRCC), prognosis, tumor microenvironment, nonnegative matrix factorization (NMF)

## Abstract

**Background:** EMILIN2 is a platelet-associated elastin that regulates angiogenesis. It has recently been found to play an essential role in various tumors. Nevertheless, the mechanism of action of EMILIN2 in clear cell renal cell carcinoma (ccRCC) remains unclear.

**Methods:** Samples from 33 cancers were obtained from UCSC Xena and The Cancer Genome Atlas (TCGA) database. The relationship between EMILIN2 expression and the clinicopathological characteristics and immune infiltration of ccRCC was investigated. Nonnegative matrix factorization (NMF) was used to classify ccRCC patients. A multigene risk prediction model of ccRCC was constructed using LASSO regression and multivariate regression analysis. A nomogram survival probability prediction map and calibration curve were constructed based on clinical information.

**Results:** EMILIN2 is significantly overexpressed in ccRCC, a phenomenon that is associated with poor prognosis. Meanwhile, EMILIN2 expression is closely related to tumor immune infiltration in ccRCC. Patients with clear cell renal cell carcinoma were divided into two subtypes using NMF, with subtype 2 showed poor prognosis. Next, we established a risk score model for ccRCC based on the common differentially expressed genes (DEGs) between subtypes and groups based on EMILIN2 expression. The results indicated poor prognosis in the high-risk group in the training set and were confirmed in the validation set.

**Conclusion:** Our findings suggest that EMILIN2 expression is closely associated with immune infiltration in ccRCC. EMILIN2 expression is negatively correlated with the prognosis of ccRCC patients. Here, we developed a tool that could predict the prognosis of ccRCC patients.

## Introduction

Clear cell renal cell carcinoma is the most common form of renal cancer. The occurrence of ccRCC is associated with multiple factors, such as smoking, drugs, obesity and others (Koul et al., 2011). Patients with clear cell renal cell carcinoma have a high probability of tumor metastasis. At present, there are two main treatment methods for ccRCC: surgical treatment and immunotherapy. If the disease is confined to the kidney in the early stage, surgical resection may be considered (Petejova and Martinek, 2016). If the tumor exhibits metastases, whether local or systemic, the survival rate is dramatically reduced (Siegel et al., 2019). However, the form of cytotoxic chemotherapy currently used is not very effective for the treatment of ccRCC. Molecular targeted drugs such as tyrosine kinase inhibitors (TKIs) and vascular endothelial growth factor receptor (VEGFR) inhibitors are often used to treat ccRCC patients because of the strong dependence of the tumor on angiogenesis (Vuong et al., 2019).

The degree of immune infiltration in ccRCC is also high among various cancers. Because the complex tumor microenvironment plays a critical role in the treatment of patients, the study of the tumor microenvironment is closely related to the identification of relevant factors and the development of drugs. Commonly used immune checkpoint inhibitors (ICI) that block the inhibitory receptor on PD-1/PD-L1 or CTLA-4 T cells have been shown to be very effective against this cancer type (Motzer et al., 2015; Motzer et al., 2018).

EMILIN2 is located on the short arm of human chromosome 18, encoding an extracellular matrix glycoprotein with a relative molecular mass of 116 kD and five protein domains: C-terminal C1q domain, proline-rich domain, collagenous domain, coiled-coil domain, and N-terminal cysteine-rich domain (EMI domain) (Bressan et al., 1993; Colombatti et al., 2000). EMILIN2 has been shown to bind to EMILIN1. Both are elastin microfiber interface proteins that play an important role in imparting elasticity to tissue and blood vessels (Colombatti et al., 2000). Several studies have shown that EMILIN2 is associated with anti-PD1 therapy in melanoma (Fejza et al., 2021). In gastric cancer, EMILIN2 regulates the proliferation of cancer cells through apoptosis (Andreuzzi et al., 2020). The loss of EMILIN2 expression leads to defective angiogenesis (Paulitti et al., 2018).

Although large body of evidence indicates that EMILIN2 is associated with tumor immunity and angiogenesis in various cancers, the role of EMILIN2 in ccRCC remains elusive. Investigation into the role of EMILIN2 in ccRCC may help improve our understanding of the occurrence, progression, and metastasis of ccRCC and aid the development of new therapeutic strategies. In this study, we identified EMILIN2 as an independent prognostic factor in ccRCC patients. High EMILIN2 expression was found to predict poor prognosis in ccRCC patients. EMILIN2 expression is strongly associated with cancer progression in ccRCC patients. Immune checkpoint, immune infiltration and enrichment analyses revealed that EMILIN2 expression was significantly associated with the immunity of ccRCC patients. And we developed a comprehensive prognostic model of ccRCC with good performance.

## Methods and materials

### Data access

We obtained the RNA-sequencing data and clinical data of 33 types of cancer patients from UCSC Xena (https://xenabrowser.net/datapages/) and TCGA database (https://www.cancer.gov/about-nci/organization/ccg/research/structural-genomics/tcga), including age, gender, tumor stage and survival data. The gene expression dataset format is TPM. The data set of ccRCC contained 531 cancer tissue samples and 72 adjacent tissue samples. The immune, matrix and ESTIMATE scores of patients were calculated by ESTIMATE (Yoshihara et al., 2013) (https://bioinformatics.mdanderson.org/estimate/rpackage.html).

### Determination of tumor-infiltrating immune cells in clear cell renal cell carcinoma

We calculated the respective scores of 27 immune cells in ccRCC patients by single sample gene set enrichment analysis. These cell types are activated CD4^+^ T cell, activated CD8^+^ T cell, activated dendritic cell, CD56 bright natural killer (NK) cell, central memory CD4^+^ T cell, central memory CD8^+^ T cell, NK cell, NK T cell, type 1 T helper cell, type 17 T helper cell, CD56 dim NK cell, immature dendritic cell, macrophage, myeloid derived suppressive cell (MDSC), neutrophil, plasmacytoid dendritic cell, regulatory T cell (Treg), type 2 T helper cell, activated B cell, eosinophil, gamma delta T cell, immature B cell, mast cell, memory B cell, monocyte and T follicular helper cell (Foroutan et al., 2018).

### Gene set enrichment analysis

In this analysis, the clusterProfiler package was used to analyze the pathway changes between patients. Patients were divided into high and low groups according to the median EMILIN2 expression by limma, and the DEGs were obtained (Ritchie et al., 2015). Then the clusterProfiler package was used to perform KEGG and GO enrichment analysis for these DEGs (Wu et al., 2021).

### Molecular subtyping was performed using a non-negative matrix factorization (NMF) algorithm

We performed Cox regression analysis using the CancerSubtype package to identify the gene sets of 27 immune cell types obtained from the TISIDB (Xu et al., 2017) (http://cis.hku.hk/TISIDB/data/download/CellReports.txt). Following this, the NMF clustering algorithm was used to cluster ccRCC samples based on the screened genes (Brunet et al., 2004). The NMF package was used for NMF analysis. In the NMF method, the standard “Brunet” option was selected, and data were subjected to 50 iterations. The number of clusters “k” ranged from 2 to 10. Finally, the optimal number of clusters was found to be 2. The DEGs between molecular subtypes were identified using the limma package.

### Development and validation of the risk scoring model

After the common DEGs between the molecular subtypes of ccRCC and between high- and low-EMILIN2 expression groups were identified, ccRCC samples were divided into the training and validation sets in an 8:2 ratio. Following this, we calculated the risk score by LASSO regression and multivariate regression analysis based on “risk scores = $\sum coef*\mathrm{Exp}\left(genes\right)$ .” Based on the findings, a six-gene prognostic model was established. We then used the ROC curve to assess the ability of this feature to predict patient survival in the high-risk and low-risk groups.

We selected the immunohistochemical staining images of these model genes in normal tissues and ccRCC tissues from the Human Protein Atlas database (http://www.proteinatlas.org).

### Construction of nomogram

To assess the predictive accuracy and importance of risk models and clinical characteristics, we analyzed the predictive relationships among age, sex, and TNM stage. And the importance of examining the RiskScore factors from one or more perspectives. Then the nomogram model including age, gender, TNM stage and risk score was constructed. Nomogram calibration plots were used to compare predicted survival events at 1, 3, and 5 years with actual observations.

### Cell lines and quantitative polymerase chain reaction

The human ccRCC cell line (786-O) and human renal epithelial cell line (293T) used for reverse-transcription-quantitative polymerase chain reaction (RT-qPCR) in this study were purchased from Procell (Wuhan, China). Total RNA was isolated using Trizol (Takara, Japan). HiScript III RT SuperMix for qPCR (+gDNA Wiper) (Nanjing, China) was used for cDNA synthesis. qPCR was performed using the SYBR Green assay according to the manufacturer’s protocol. Then the mRNA expressions of TNNT1, SAA1, IL20RB, COL22A1, B3GALT5, and C10orf99 were analyzed.

The following primers were used for RT-qPCR:TNNT1, forward:5′-TGATCCCGCCAAAGATCCC-3′; TNNT1, reverse:5′- TCT​TCC​GCT​GCT​CGA​AAT​GTA-3′; SAA1, forward:5′- GAT​CAC​CGA​TGC​CAG​AGA​GA-3′; SAA1, reverse:5′- TTT​GTA​TCC​CTG​CCC​TGA​GG-3′; IL20RB, forward:5′- GGC​CAC​TGT​GCC​ATA​CAA​C-3′; IL20RB, reverse:5′- TCT​TTG​GTG​ATC​TCC​ATC​CCA -3′; COL22A1, forward:5′- CCT​AGC​GTT​CGT​GTA​GAA​GGA-3′; COL22A1, reverse:5′- CCC​ATC​CGT​ACA​TAG​GAA​CTC​T-3′; B3GALT5, forward:5′- AAG​CTC​CCA​GAT​ACA​GAC​TGC-3′; B3GALT5, reverse:5′- TGG​TCC​ACC​TCT​TTC​GTT​TCC-3′; C10orf99, forward:5′- CCG​GTC​ACA​GCT​ACA​AAT​CC-3′; C10orf99, reverse:5′- TCA​GGA​GGC​TAG​GAA​GGG​AT-3′; G A P D H, f o r w a r d: 5′-GGA​GCG​AGA​TCC​CTC​CAA​AAT-3′; and GAPDH, reverse:5′-GGCTGTTGTCATACTTCTCATGG-3′.

### Statistical analysis

All data analyses were based on R software (software version 4.0.2). We then used Student’s *t*-test to calculate the significance of the difference between the two groups. *p*-value greater than 0.05 was considered significant.

## Result

### Elastin microfibril interfacer 2 is expressed at high levels in clear cell renal cell carcinoma and indicates a poor prognosis

**FIGURE 1:**
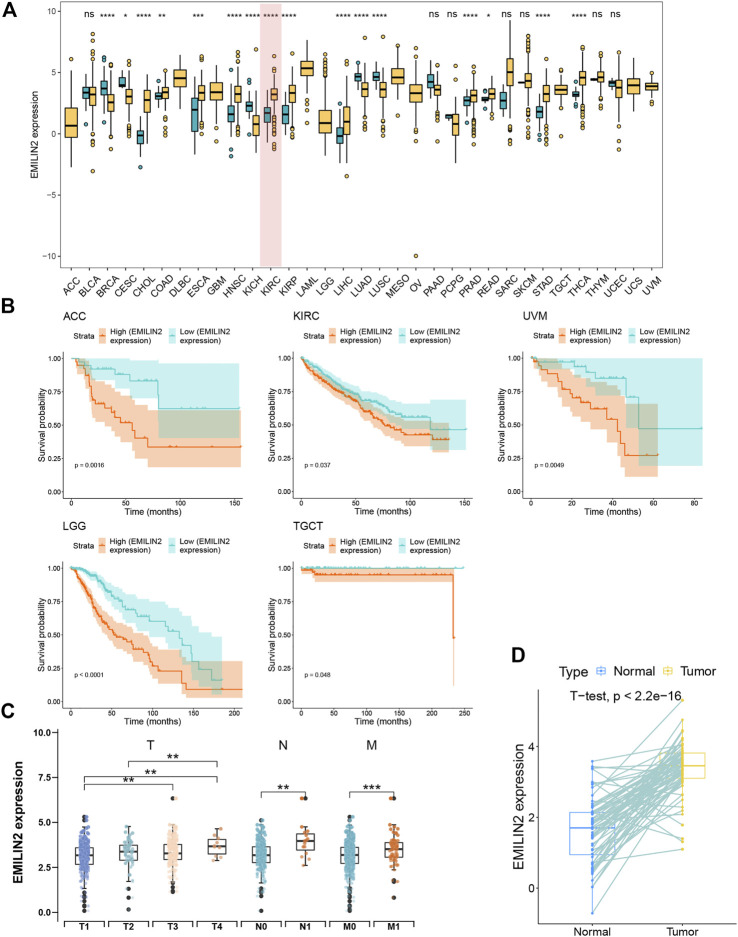
Pan-cancer analysis and expression analysis of EMILIN2 **(A)** EMILIN2 expression in different cancers, *p < 0.05, **p < 0.01, ***p < 0.001, ns refers to not significant. **(B)** The survival map of EMILIN2 in different cancers. **(C)** EMILIN2 expression in different tumor stages, *p < 0.05, **p < 0.01, ***p < 0.001, ns refers to not significant. **(D)** EMILIN2 expression in tumor tissues and its paired adjacent tissues, *p < 0.05, **p < 0.01, ***p < 0.001, ns refers to not significant.

To the effect of EMILIN2 on various cancer types, we analyzed the EMILIN2 expression in cancer tissues and adjacent tissues in 33 types of cancer using data from TCGA. EMILIN2 was found to be expressed differentially in various cancer types (*p* < 0.05). For example, EMILIN2 expression was significantly up-regulated in cholangiocarcinoma, colon adenocarcinoma, and ccRCC (Figure 1A). Concurrently, we analyzed the prognosis of EMILIN2 in these 33 cancers. EMILIN2 was found to have prognostic significance in five cancers. For example, high EMILIN2 expression led to poor prognosis in adrenocortical carcinoma, ccRCC, brain bower grade glioma, testicular germ cell tumors and uveal melanoma (*p* < 0.05) (Figure 1B). Interestingly, EMILIN2 was differentially expressed only in ccRCC (*p* < 0.0001) and showed prognostic power. In order to study the relationship between ELIMIN2 expression and tumor stage, we analyzed the EMILIN2 expression in ccRCC patients using TNM stage information. The results showed that the EMILIN2 expression was significantly different in different tumor pathological stages. For example, EMILIN2 expression is significantly higher in patients with distant metastasis (n.s., not significant; *p < 0.05, **p < 0.01, ***p < 0.001) (Figure 1C). These results indicate that the EMILIN2 expression is closely related to tumor progression. In addition, we analyzed the EMILIN2 expression in paired ccRCC and adjacent normal tissues, and found that the EMILIN2 expression was significantly higher in tumor tissue samples (Figure 1D).

### Elastin microfibril interfacer 2 is associated with multiple immune checkpoints

**FIGURE 2:**
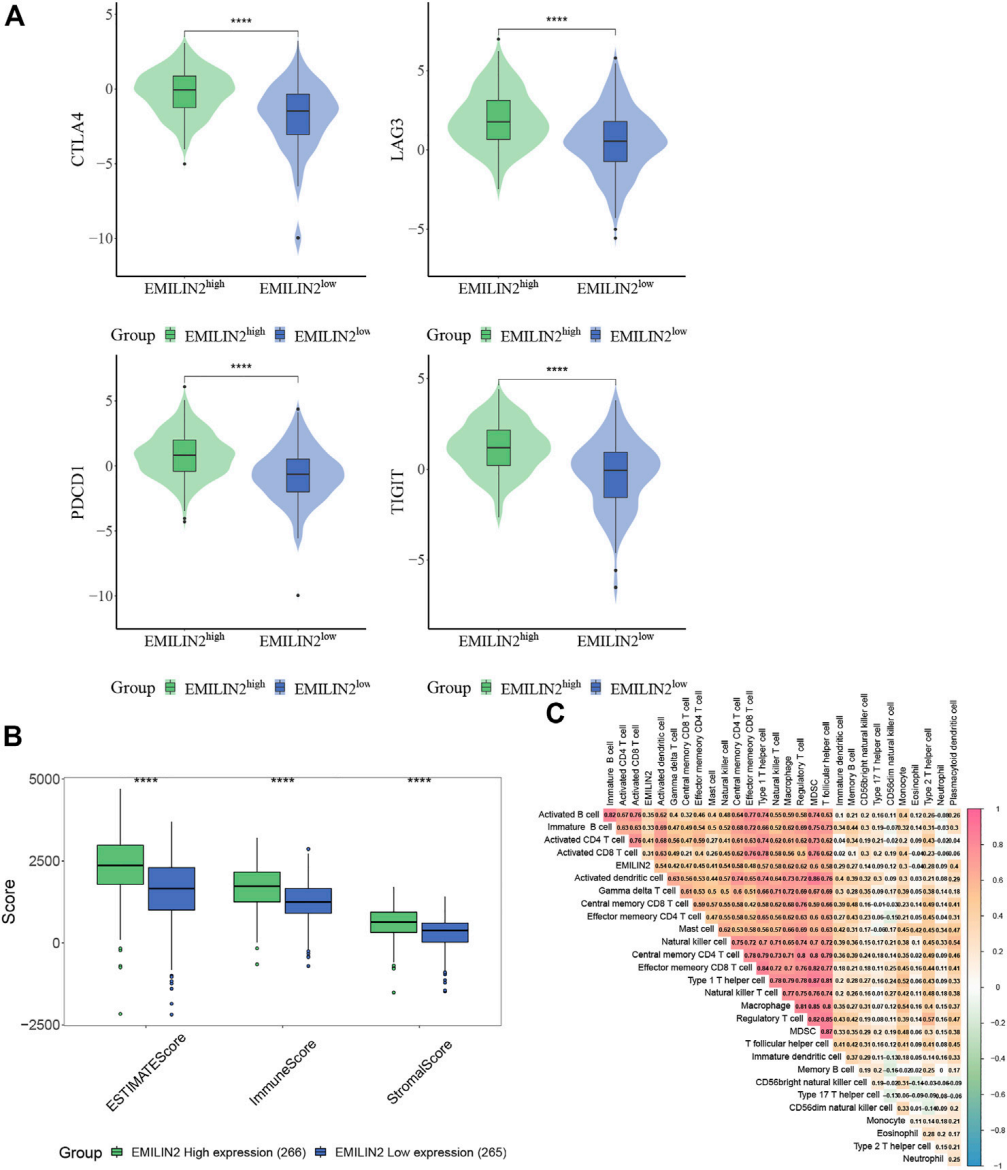
EMILIN2 is closely associated with immune infiltration in ccRCC **(A)** The EMILIN2 expression in high and low expression groups of different immune checkpoints, *p < 0.05, **p < 0.01, ***p < 0.001, ns refers to not significant. **(B)** Immune, Stromal, and ESTIMATE Scores of EMILIN2 high and low expression groups, *p < 0.05, **p < 0.01, ***p < 0.001, ns refers to not significant. **(C)** Correlation analysis of EMILIN2 and 27 kinds of immune cells.

The study of immune checkpoint is crucial for the treatment of cancer. To analyze the relationship between EMILIN2 and immune checkpoints, ccRCC patients were divided into high and low groups according to the median EMILIN2 expression. The results showed that several checkpoints including CTLA-2, PDCD1, LAG3, and TIGIT were significantly overexpressed in the EMILIN2 high expression group (Figure 2A). This indicates that the group with high EMILIN2 expression may have a better effect on immunotherapy.

### Analysis of tumor immune infiltration with elastin microfibril interfacer 2

The tumor microenvironment is composed of immune cells and stromal cells in addition to tumor cells. The complexity of tumor microenvironment has a significant impact on tumor treatment. To analyze the relationship between ELIMIN2 and tumor microenvironment, we calculated the immune score, stromal score, and ESTIMATE score of ccRCC patients by ESTIMATE. The results showed that the patients with high ELIMIN2 expression had immune score, stromal score, and ESTIMATE score (*p* < 0.0001) (Figure 2B). These evidences indicate that EMILIN2 is closely related to the tumor microenvironment.

To further analyze which immune cells were associated with EMILIN2, we calculated the scores of 27 types of immune cells in ccRCC patients by ssGSEA and found that EMILIN2 was significantly positively correlated with all 27 types of immune cells (*p* < 0.05) (Figure 2C). These evidences suggest that EMILIN2 is closely related to immune infiltration in ccRCC patients.

### Patients with clear cell renal cell carcinoma can be categorized into two subtypes based on the findings of the nonnegative matrix factorization algorithm

**FIGURE 3:**
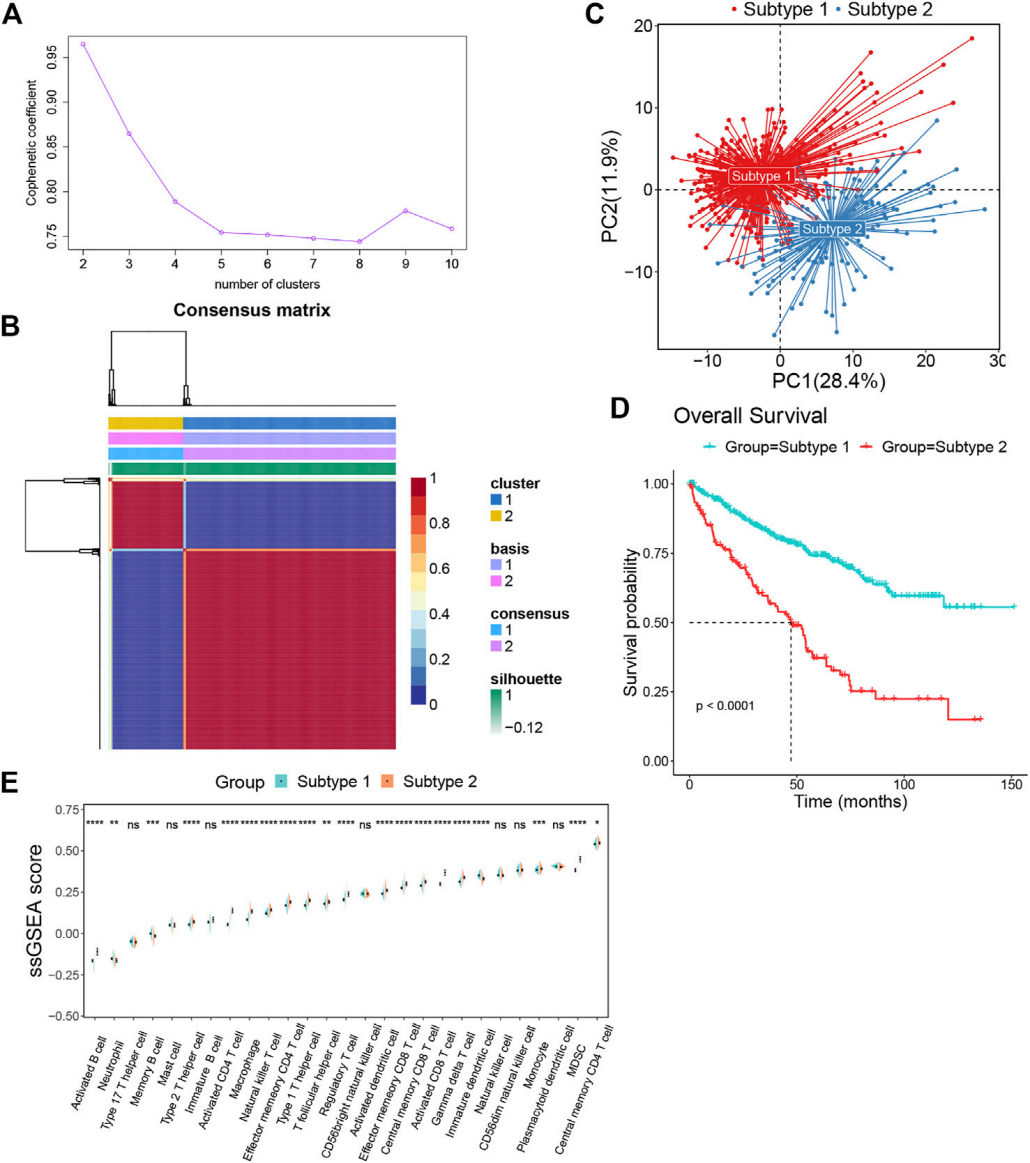
The ccRCC patients were divided into two subtypes by NMF algorithm **(A)** The best rank value was selected according to the Cophenetic value. **(B)** Cluster heatmap of two clusters (k = 2). **(C)** Principal component analysis of two subtypes. **(D)** The survival map of the two subtypes. **(E)**The differences in immune cell fractions among subtypes, *p < 0.05, **p < 0.01, ***p < 0.001, ns refers to not significant.

We obtained the gene sets of 27 immune cell types (749 genes in total) that showed a significant positive correlation with EMILIN2. Next, we conducted multivariate regression analysis using the CancerSubtypes package for feature screening and identified 285 genes. Based on TCGA data, ccRCC patients were classified according to the genes expressed using the NMF package. The best value of K was 2 (Figure 3A). Finally, ccRCC patients were divided into two subtypes (Figure 3B). PCA was performed to evaluate the results of NMF typing, results indicated that the two subtypes showed significant differences (Figure 3C). Moreover, a significant difference was observed between in the survival of patients with the two subtypes of cancer. Patients with subtype 1 ccRCC had better overall survival, whereas patients with subtype 2 ccRCC had poor overall survival (Figure 3D).

To investigate the differences between subtypes 1 and 2, we analyzed immune cell infiltration in these two subtypes. Most immune cells, such as activated B cells, type 2 T helper cells, activated CD4^+^ T cells, macrophages, natural killer T cells, effector memory CD4^+^ T cells, type 1 T helper cells, t follicular helper cells, regulatory T cells, activated dendritic cells, effector memory CD8^+^ T cells, central memory CD8^+^ T cells, activated CD8^+^ T cells, gamma delta T cells, monocytes, MDSCs and central memory CD4^+^ T cells were more enriched in subtype 2 than subtype 1. Meanwhile, only a few immune cells were enriched in subtype 1, such as neutrophils, memory B cells and immature dendritic cells (Figure 3E).

### Construction of a risk-score model for clear cell renal carcinoma

**FIGURE 4:**
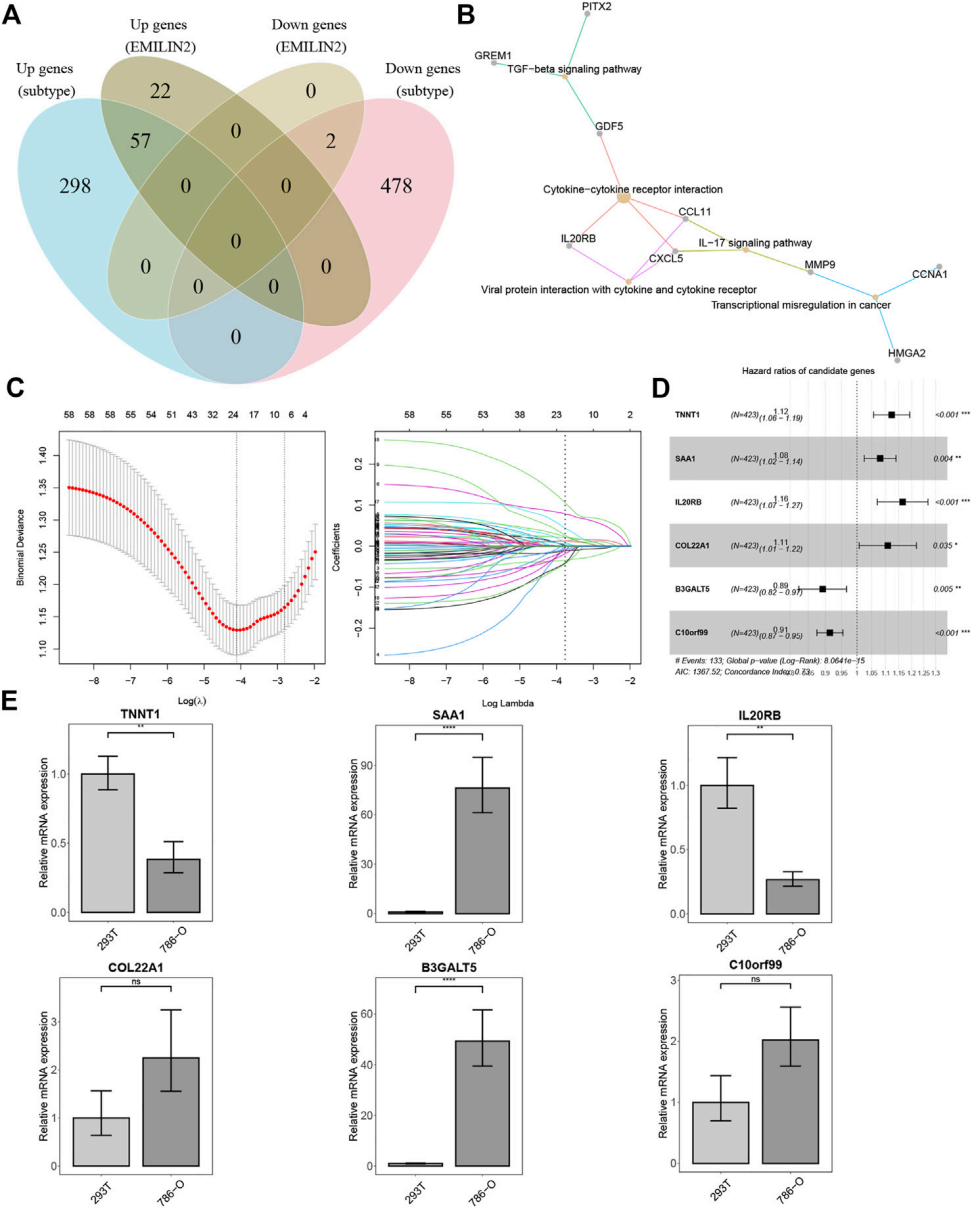
Establishment of risk score model and qPCR results **(A)** VEEN diagram of different DEGs. **(B)** KEGG enrichment analysis of 59 intersection genes. **(C)** The best *λ* value is obtained by ten-fold cross validation. **(D)** The forest map of risk model genes. **(E)** qPCR results of risk model genes, *p < 0.05, **p < 0.01, ***p < 0.001, ns refers to not significant.

We analyzed the DEGs between the two subtypes and identified 355 upregulated and 478 downregulated genes. In addition, we identified the DEGs between patients in high and low expression groups according to the median EMILIN2 expression. Seventy-nine genes were upregulated and two genes were downregulated. Next, we evaluated the intersection of DEGs in the high and low EMILIN2 expression groups and the DEGs in the two subtypes of ccRCC to obtain a total of 59 genes (Figure 4A). These genes are associated with TGF-beta signaling pathway, cytokine-cytokine receptor interaction, viral protein interaction with cytokine and cytokine receptor, IL-17 signaling pathway and transcriptional misregulation in cancer (Figure 4B). Next, after excluding patient samples without survival data, 529 ccRCC patients were categorized in a training set and a validation set in an 8:2 ratio.

In the training set, we used LASSO regression to screen genes closely associated with patient prognosis among the 59 genes. The overfitting effect was overcome by ten-fold cross-validation, and the best *λ* value obtained was 0.016 (Figure 4C). The hazard ratios (HR) of TNNT1, SAA1, IL20RB, and COL22A1 were greater than 1, suggesting that these were risk genes. The HR values of B3GALT5 and C10orf99 were less than 1, suggesting these were protective genes (Figure 4D). We calculated the risk score for each ccRCC patient using the following formula:Risk score=(0.11597*TNNT1)+(0.07741*SAA1)+(0.15255*IL20RB)+(0.10302*COL22A1)+(−0.11426*B3GALT5)+(−0.09019*C10orf99). We used RT-qPCR to analyze the mRNA expression levels of the six genes in 786-O cells (derived from the renal epithelial cells of ccRCC patients) and 293T cells (human renal epithelial cells). TNNT1 and IL20RB had higher mRNA expression levels in 293T cells, SAA1 and B3GALT5 had higher mRNA expression levels in 786-O cells, and C10orf99 and COL22A1 showed no significant difference in mRNA expression levels in the 2 cell types (Figure 4E).

We analyzed the protein expression levels of the six model genes using the Human Protein Atlas database. TNNT1, COL22A1, and C10orf99 showed no significant difference in expression between cancer tissues and normal tissues, B3GALT5 expression was relatively high in cancer tissues, whereas SAA1 and IL20RB did not show protein expression (Supplementary Figures S1A–D).

### Prognostic value of the risk score model

**FIGURE 5:**
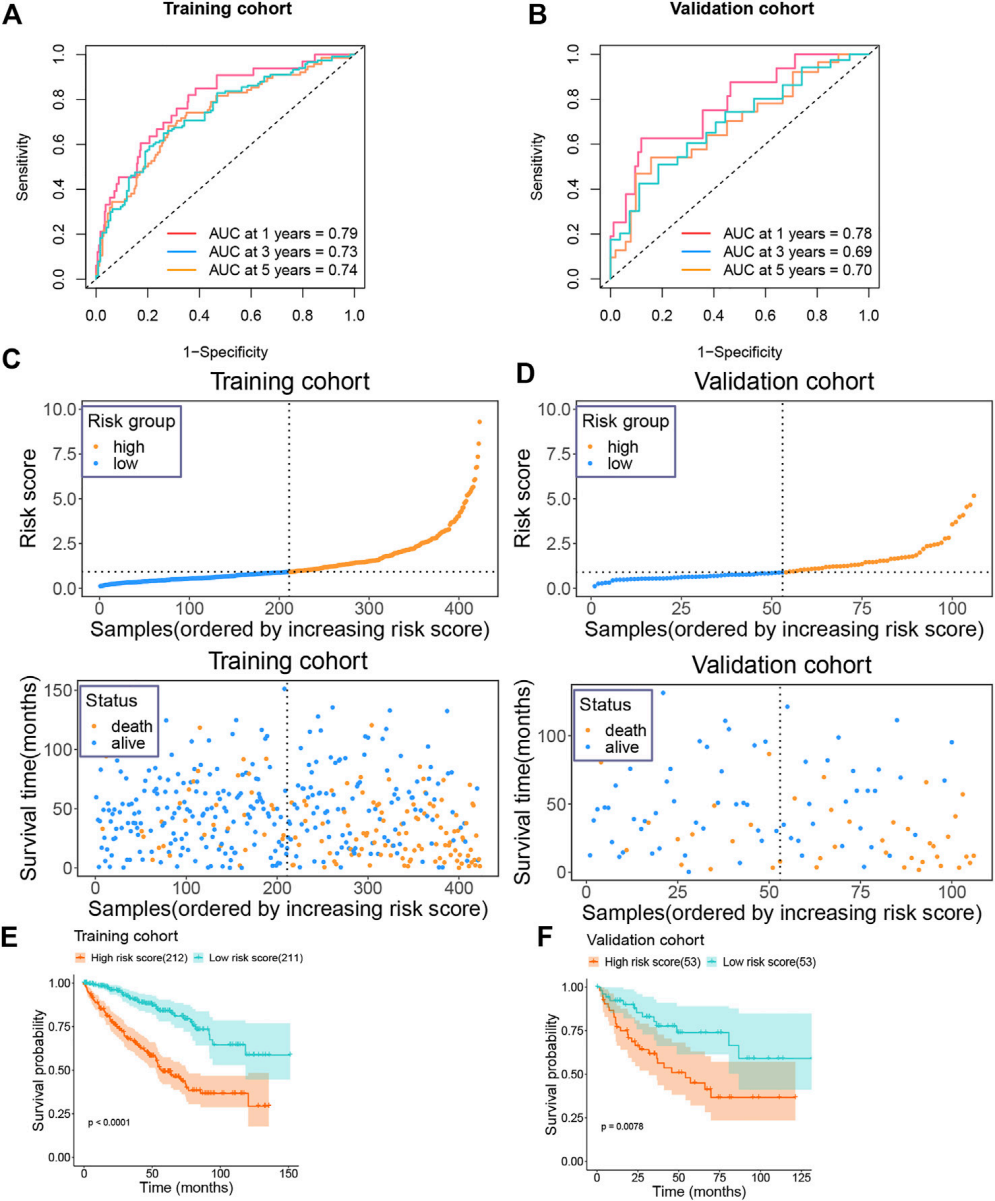
Prognostic value of risk model in training and validation groups **(A)** The AUC value of the risk model in the training group. **(B)** The AUC value of the risk model in the validation group. **(C)** Risk score and survival status of the patients in the training group. **(D)** Risk score and survival status of the patients in the validation group. **(E)** Survival plot of the high and low risk groups in the training group. **(F)** Survival plot of the high and low risk groups in the validation group.

Following this, we calculated the ROC value of the model in the training set. The model showed good risk prediction potential at 1, 3, and 5 years. The AUC value of the 1-year prediction was 0.79, that of the 3-year prediction was 0.73, and that of the 5-year prediction was 0.74 (Figure 5A). Similarly, in the validation set, the AUC of the model was 0.78 for the 1-year prediction, 0.69 for the 3-year prediction, and 0.7 for the 5-year prediction (Figure 5B).

We then categorized the patients in the training set into high-risk and low-risk groups according to their median risk score. As the risk score increased, the number of patients who died also increased (Figure 5C). Similarly, the data in the validation set showed consistently changes (Figure 5D). In addition, we also analyzed the survival of patients in the high-risk and low-risk groups. Patients in the high-risk group showed a very poor prognosis in the training set (Figure 5E). In the validation set, patients in the high-risk group also showed a poor prognosis (Figure 5F).

### The nomogram was constructed and verified by combining clinical data

**FIGURE 6:**
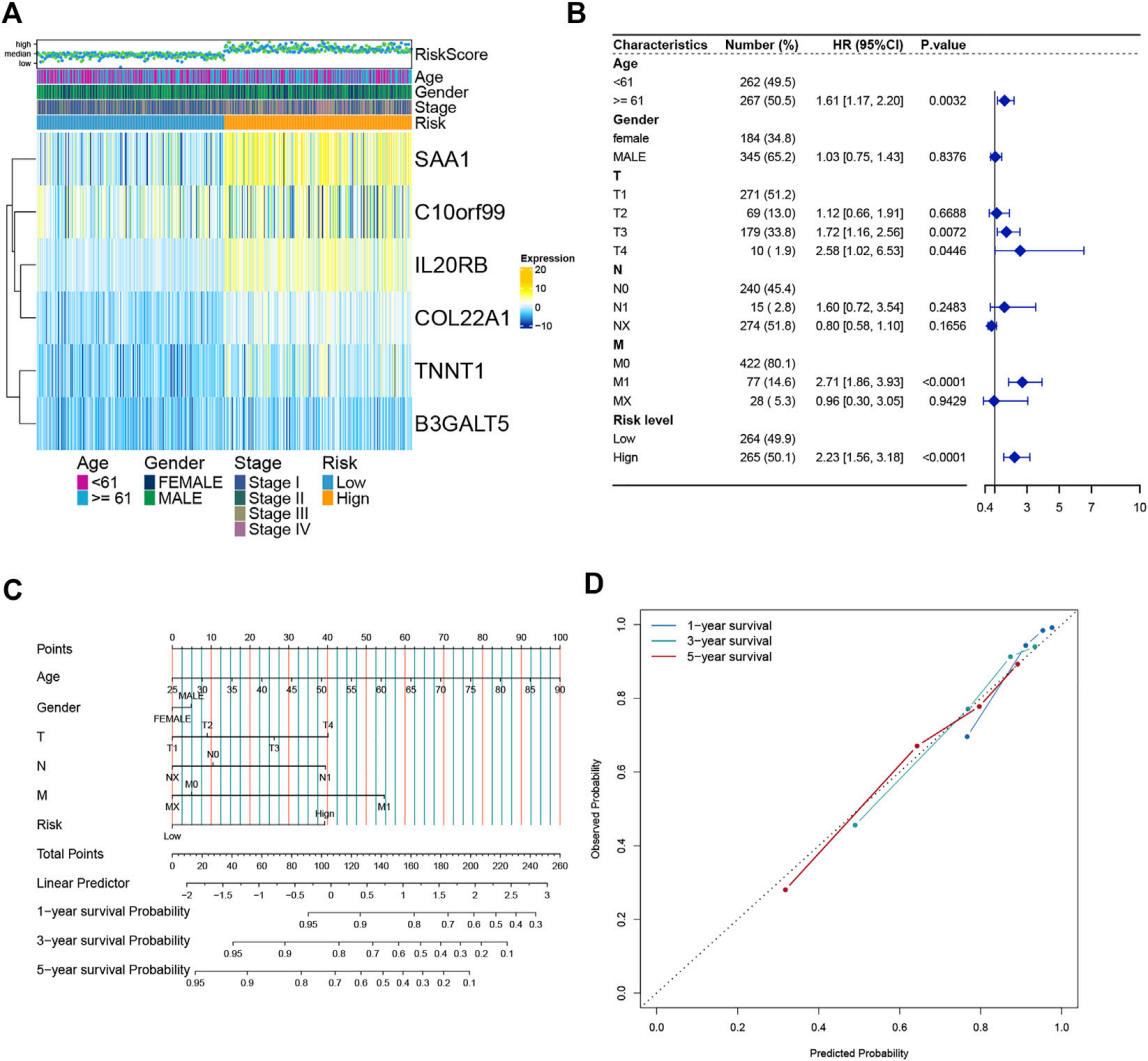
Establishment and verification of the Nomogram model **(A)** Heatmap of the expression of six model genes in all ccRCC patients. **(B)** Forest map of Risk score and clinical features. **(C)** Nomograms to predict 1 -, 3 -, and 5-year OS in ccRCC patients. **(D)** Calibration curves of the nomogram.

We analyzed the expression of six genes from the risk score model in all ccRCC patients. These six genes were found to be expressed differently in patients with different risk scores (Figure 6A). We then performed multivariate regression analysis by combining the risk scores of patients with clinical data to explore the association between the risk scores and clinical characteristics. Older age, advanced T stage, distant metastasis of the tumor, and a high risk score were identified as significant risk factors (Figure 6B). Previous analyses showed that the risk score value was significantly associated with patient survival. To develop a more accurate clinical prediction method for ccRCC patients, we constructed a comprehensive nomogram model combining the patient’s risk score, gender, TNM stage, and age, which could be used to predict the 1-year, 3-year, and 5-year survival of patients (Figure 6C). Moreover, according to the results of the calibration map for patient survival prediction, the prediction results of the nomogram were in good agreement with the actual observation (Figure 6D). In conclusion, our analysis revealed that the constructed nomogram showed good performance in predicting the survival of ccRCC patients.

### Pathway enrichment analysis

**FIGURE 7:**
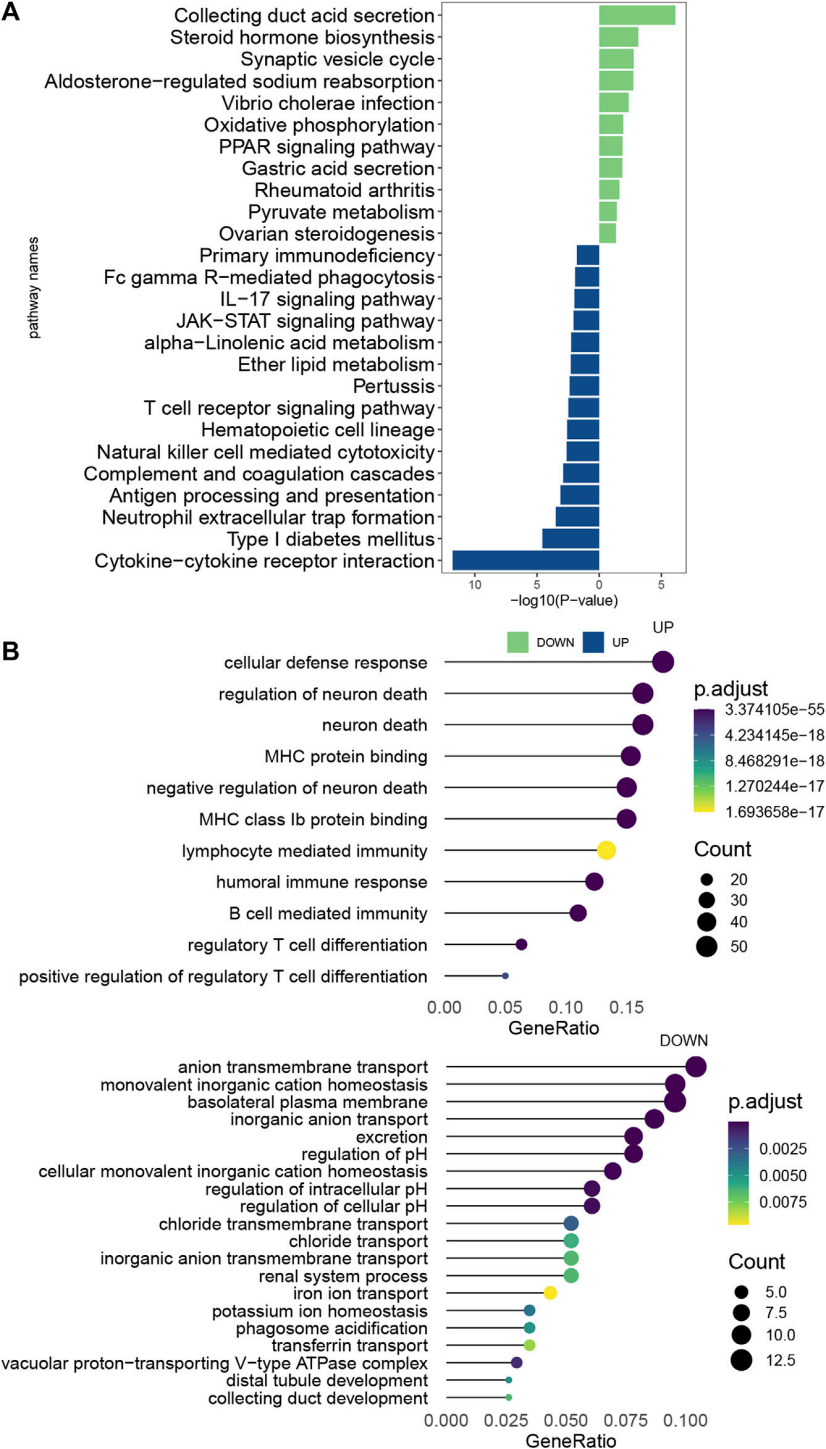
Pathway enrichment analysis of EMILIN2 **(A)** Differential pathways between KEGG-enriched EMILIN2 high and low expression groups. **(B)** Differential pathways between GO-enriched EMILIN2 high and low expression groups.

The ccRCC samples (cancer tissues and adjacent tumor samples) were divided into high and low expression groups according to the median expression of EMILIN2. Using the limma package, 366 DEGs were obtained, of which 235 genes were upregulated and 131 genes were downregulated. To explore the potential functions of these DEGs, we analyzed the biological pathways that these genes are associated with using the clusterProfiler package. In KEGG pathway analysis, cytokine-Cytokine_receptor_interaction, TCR signaling pathway, IL-17 signaling pathway, and natural killer cell-mediated cytotoxicity were significantly upregulated (Figure 7A). GO analysis revealed that these DEGs were significantly enriched in cellular defense response, regulatory T-cell differentiation, and B-cell-mediated immunity in biological processes. Moreover, these DEGs were significantly enriched in the basolateral plasma membrane in cellular components and significantly enriched in MHC class IB protein binding and MHC protein binding in molecular function (Figure 7B). This finding suggests that DEGs are associated with many important immune processes associated with T cells and B cells and may play an important role in tumor immunity in patients. The finding also indicates that EMILIN2 expression is closely associated with the immunity of patients. In addition, IL-17 signaling was found to affect angiogenesis, suggesting that EMILIN2 expression may be associated with angiogenesis in ccRCC.

## Discussion

Clear cell renal cell carcinoma, the most common type of renal cancer, is a tumor highly dependent on vascular survival. At present, surgical treatment and immunotherapy are the major approaches used to treat patients with this cancer type (Siegel et al., 2019). Several immunotherapeutic drugs have been developed for treating ccRCC (Motzer et al., 2018) (Motzer et al., 2015). However, owing to the complexity of the tumor microenvironment, killing and removing tumor cells by immunotherapy is a complex process, and achieving good effectiveness is not feasible. Therefore, continuous exploration of the tumor microenvironment and the search for better immunotherapeutic agents remains a major challenge. Identifying new novel biomarkers that are closely related to patient immunity and survival is also of great significance.

In this study, bioinformatics analysis showed that EMILIN2, a gene that regulates angiogenesis, was significantly upregulated in ccRCC patients and had prognostic potential. Concurrently, EMILIN2 expression was found to be closely related to the immune status of ccRCC patients. EMILIN2 expression was found to be significantly correlated with multiple immune markers and immune cells. We then obtained the gene sets of these immune cells and performed the molecular typing of ccRCC patients using the NMF algorithm. Patients with clear cell renal cell carcinoma were grouped according to EMILIN2 expression, and the intersection of genes showing differential expression in different groups and subtypes was obtained. Based on this, a six-gene model was constructed and verified by LASSO regression and multivariate regression analysis. We then developed and validated a comprehensive nomogram model by combining risk scores and clinical data.

We first analyzed the EMILIN2 expression in 33 cancer types and its ability to predict patient survival. EMILIN2 showed a significant differential expression in ccRCC and indicated poor prognosis. Thus, EMILIN2 expression was closely related to the prognosis of ccRCC. Following this, we investigated the association between EMILIN2 and immune checkpoints, and found that the group with high EMILIN2 expression showed the up-regulation of immune checkpoints. This implies that EMILIN2 expression has some potential effect on the immunotherapy of patients. We then calculated the immune, stromal and ESTIMATE scores of ccRCC patients using ESTIMATE, which is often used to study the tumor microenvironment and explore immune and stromal cells in the tumor microenvironment of different patients. Patients with high EMILIN2 expression also showed a significant increase in immune and stromal scores. This indicates that EMILIN2 expression affects the tumor microenvironment in ccRCC. The study of the complex tumor microenvironment is of great significance in the treatment of patients with tumor. These findings suggest that EMILIN2 may play an important role in tumor development, metastasis and immune response.

To explore the immune cells EMILIN2 might be associated with, we used ssGSEA to calculate the scores for 27 types of immune cells in ccRCC patients. Interestingly, EMILIN2 expression was positively correlated with the scores of 27 immune cell types, especially T cells. Thus, EMILIN2 expression affects the extent of infiltration of various immune cells in tumors. The tumor infiltration level of immune cells is an important index for prognostic judgment and evaluating therapeutic effects. Therefore, EMILIN2 has extremely important research value in the study of immune invasion of tumors. Next, we identified DEGs between the high and low EMILIN2 expression groups. KEGG pathway enrichment results revealed that these DEGs were significantly enriched in immune and vascular pathways, such as the TCR signaling pathway and IL-17 signaling pathway. Many studies have shown that the TCR signaling pathway plays an important role in tumor immunity, and the downstream signaling pathway mediated by the TCR signaling pathway plays a key role in promoting the anti-tumor immunity of CD8^+^ T cells. The interaction of T cell receptors with MHC antigenic peptide complexes leads to changes in T cells at the molecular and cellular levels and mediates the activation of various genes (Shah et al., 2021). The IL-17 signaling pathway is associated with angiogenesis, and IL17 is an important pro-inflammatory factor. IL-17 can promote the activation of the STAT3 signal transduction pathway through the intermediate mediator IL-6 in tumor cells. This leads to the upregulation of angiogenic factors, thereby promoting tumor angiogenesis (Yu et al., 2007; Lavecchia et al., 2011; Zhang et al., 2012). Previous studies have shown that EMILIN2 regulates angiogenesis. For example, EMILIN2 expression in gastric cancer was found to be related to angiogenesis (Andreuzzi et al., 2020). Collectively, these results suggest that the DEGs are involved in various immune processes and may affect angiogenesis. This indicates that EMILIN2 expression is significantly associated with tumor immunity.

We divided ccRCC patients into two subtypes by screening signature genes from the gene set of 27 immune cell types. We identified 835 DEGs while analyzing differences in the expression between subtypes.

To further investigate the factors affecting the survival of ccRCC patients, we studied the intersection of the DEGs with the DEGs in the high and low EMILIN2 expression groups. These DEGs was associated with the TGF−beta signaling pathway, Cytokine−cytokine receptor interaction, Viral protein interaction with cytokine and cytokine receptor, IL−17 signaling pathway and Transcriptional misregulation in cancer were significantly correlated. In the training set, we constructed a 6-gene risk score model based on the 59 genes by combining lasso regression and multivariate regression. We then confirmed the predictive ability in the validation set. TNNT1, SAA1, IL20RB, and COL22A1 are risk factors for ccRCC, whereas B3GALT5 and C10orf99 act as protective factors when these six genes are expressed at high levels. TNNT1 was shown to be associated with various cancers, such as colorectal cancer (Chen et al., 2020). It was shown to promote the progression of colorectal cancer (Hao et al., 2020). In addition, TNNT1 may also promote the proliferation of breast cancer cells by promoting G1/S phase transition (Shi et al., 2018). SAA1 is transcriptionally activated by STAT3 and accelerates renal interstitial fibrosis by inducing ER stress (Zhang et al., 2021). Concurrently, high SAA1 expression is associated with poor prognosis in advanced renal cell carcinoma (Li et al., 2021). IL20RB overexpression can promote cell proliferation, invasion and migration of papillary renal cell carcinoma. And its overexpression can lead to a poor prognosis in patients (Cui et al., 2019). COL22A1 plays an important role in maintaining vascular homeostasis. In addition, COL22A1 mutation may be closely associated with the occurrence of intracranial aneurysms (Ton et al., 2018). High B3GALT5 expression is associated with tumor progression and the metastasis of breast cancer, and may lead to a poor prognosis for patients with breast cancer (Liao et al., 2021). C10orf99 is associated with the proinflammatory response of skin keratinocytes and affects skin barrier formation (Dainichi et al., 2022). Meanwhile, in colon cancer, C10orf99 expression may induce G1 arrest, leading to the inhibition of colon cancer cell growth (Pan et al., 2014). RT-qPCR results revealed that the mRNA expression levels of TNNT1 and IL20RB in 293T cells were higher than those in 786-O cells, indicating that TNNT1 and IL20RB expression in 786-O cells were limited compared with 293T cells. Also, RT-qPCR analysis showed that the mRNA expression levels of SAA1 and B3GALT5 in 786-O cells were higher than those in 293T cells, which may be an important factor affecting the survival of ccRCC patients. In addition, the mRNA expression levels of C10orf99 and COL22A1 in these 2 cells were not significantly different. In conclusion, these expression of these six genes is closely related to the occurrence, and progression of cancer and angiogenesis in tumors. Thus, these genes have significant research value. The findings also indicate the reliability of the risk score model. Next, a more comprehensive nomogram model was established and verified by combining the risk score with clinical information.

However, our study may have had certain limitations, primarily because of the limited number of samples. Thus, further experiments are needed to verify the role of these six model genes. Although EMILIN2 was shown to affect angiogenesis, further experimental investigation is needed to confirm whether it affects tumor immunity by regulating angiogenesis in ccRCC.

## Conclusion

The strengths of our study are the detailed analysis of the association of EMILIN2 in tumor immunity, the molecular typing of ccRCC, and the development of a 6-gene prognostic model with a high AUC value. Then a comprehensive nomogram model was established to predict the 1/3/5-year survival rate of ccRCC patients combined with clinical information. In conclusion, our study suggests that EMILIN2, a gene that regulates angiogenesis, may be a potential target and candidate marker for the treatment of ccRCC patients.

## Data Availability

Publicly available datasets were analyzed in this study. This data can be found here: https://www.cancer.gov/about-nci/organization/ccg/research/structural-genomics/tcga.

## Supplementary material

SUPPLEMENTARY FIGURE S1 Expression of TNNT1, COL22A1, B3GALT5, and C10orf99 in the Human Protein Atlas protein database.
