# Supplementary material for: EMILIN2 is associated with prognosis and immunotherapy in clear cell renal cell carcinoma
Source: Front Genet. 2022 Dec 5;13:1058207. doi: 10.3389/fgene.2022.1058207 (PMC9760906; doi:10.3389/fgene.2022.1058207)
Supplement: Supplementary file 1 [file Image1.pdf]

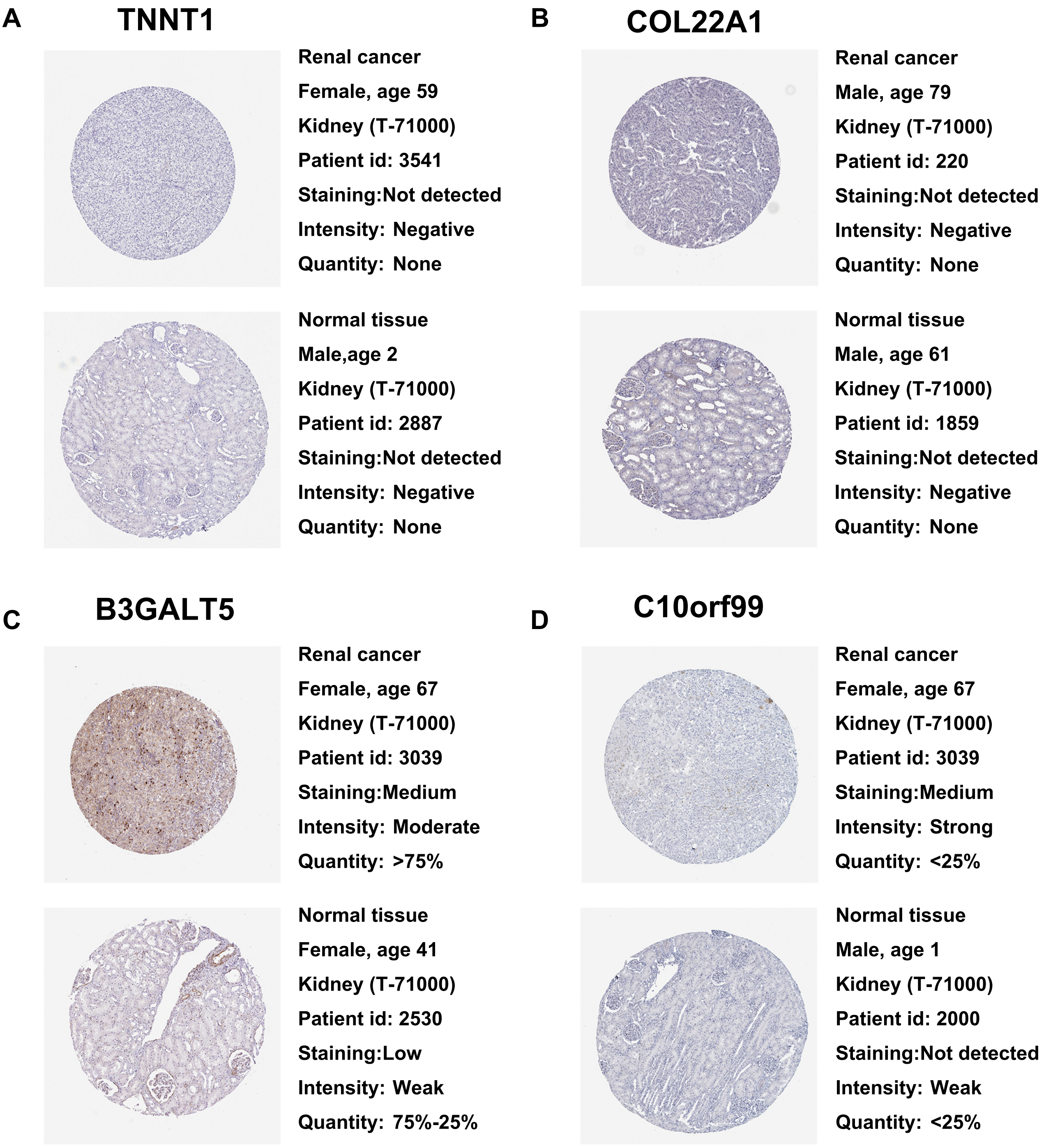
A
TNNT1
Renal cancer
Female, age 59
Kidney (T-71000)
Patient id: 3541
Staining:Not detected
Intensity: Negative
Quantity: None
Normal tissue
Male,age 2
Kidney (T-71000)
Patient id: 2887
Staining:Not detected
Intensity: Negative
Quantity: None
B
COL22A1
Renal cancer
Male, age 79
Kidney (T-71000)
Patient id: 220
Staining:Not detected
Intensity: Negative
Quantity: None
Normal tissue
Male, age 61
Kidney (T-71000)
Patient id: 1859
Staining:Not detected
Intensity: Negative
Quantity: None
C
B3GALT5
Renal cancer
Female, age 67
Kidney (T-71000)
Patient id: 3039
Staining:Medium
Intensity: Moderate
Quantity: >75%
Normal tissue
Female, age 41
Kidney (T-71000)
Patient id: 2530
Staining:Low
Intensity: Weak
Quantity: 75%-25%
D
C10orf99
Renal cancer
Female, age 67
Kidney (T-71000)
Patient id: 3039
Staining:Medium
Intensity: Strong
Quantity: <25%
Normal tissue
Male, age 1
Kidney (T-71000)
Patient id: 2000
Staining:Not detected
Intensity: Weak
Quantity: <25%
